# Microfludic Device for Creating Ionic Strength Gradients over DNA Microarrays for Efficient DNA Melting Studies and Assay Development

**DOI:** 10.1371/journal.pone.0004808

**Published:** 2009-03-11

**Authors:** Jesper Petersen, Lena Poulsen, Henrik Birgens, Martin Dufva

**Affiliations:** 1 Department of Haematology, Copenhagen University Hospital, Herlev, Denmark; 2 Department of Micro and Nanotechnology, Technical University of Denmark, Kongens Lyngby, Denmark; Brunel University, United Kingdom

## Abstract

The development of DNA microarray assays is hampered by two important aspects: processing of the microarrays is done under a single stringency condition, and characteristics such as melting temperature are difficult to predict for immobilized probes. A technical solution to these limitations is to use a thermal gradient and information from melting curves, for instance to score genotypes. However, application of temperature gradients normally requires complicated equipment, and the size of the arrays that can be investigated is restricted due to heat dissipation. Here we present a simple microfluidic device that creates a gradient comprising zones of defined ionic strength over a glass slide, in which each zone corresponds to a subarray. Using this device, we demonstrated that ionic strength gradients function in a similar fashion as corresponding thermal gradients in assay development. More specifically, we noted that (i) the two stringency modulators generated melting curves that could be compared, (ii) both led to increased assay robustness, and (iii) both were associated with difficulties in genotyping the same mutation. These findings demonstrate that ionic strength stringency buffers can be used instead of thermal gradients. Given the flexibility of design of ionic gradients, these can be created over all types of arrays, and encompass an attractive alternative to temperature gradients, avoiding curtailment of the size or spacing of subarrays on slides associated with temperature gradients.

## Introduction

Microarray analysis is generally performed using a single working condition (i.e., one hybridization or stringency washing temperature), and thus all probes must exhibit the same thermodynamic behavior in order to operate optimally in the array. At present, it is difficult to predict probe characteristics on the basis of thermodynamic models, because surface effects are normally not taken into account [1], [2]. Ideally, melting curves should be generated for each probe on an array during assay development to determine whether the probes can function together at a given stringency (temperature and buffer composition). Besides being an invaluable decision tool for assay developers, melting curves can also be of direct use in applications where high specificity is paramount (e.g., when analyzing single nucleotide polymorphisms, SNPs) [3], [4]. The most obvious advantage of using melting curves for genotyping is that the probe set does not have to be precisely matched with regard to melting temperature (*T*m), and hence A/T-rich and G/C-rich sites can be genotyped on the same array [5]. Other applications that can benefit from relaxed restriction on *T*m matching of probes are sequencing by hybridization, microRNA analysis [6], [7], tiling arrays [8], [9], “exon” arrays [10], and even gene expression profiling [11].

Curves can be obtained by increasing the temperature or decreasing the cation concentration and studying the change in signal as a function of changed stringency. Varying the temperature is the most common method to study melting despite the fact that changing cation concentration can be a technically simpler approach. Cations destabilize nucleic acid duplexes by mechanisms other than temperature; (i) reducing counterions (e.g., Na^+^) in the buffer leads to less shielding of the negatively charged backbones of the hybridized nucleic acids, which results in a stronger electrostatic repulsion between the strands and (ii) counterions form a layer on the glass surface, which shields the electrostatic effects of the surface [1], [12]. The thickness of that layer is defined by what is called a Debye layer, which is governed by the type of electrolyte and its concentration, and to a much lesser extent also by the temperature. The depth of the Debye layer varies from <1 nm (1 bp) under the high cation concentrations present during hybridization up to tens of nanometers (many bp) during stringent washing at low cation levels. Therefore, using the cation concentration as a modulator of stringency has two effects that must be taken into consideration: strand repulsion (i.e., probe/probe, probe/target, and target/target) and shielding of the surface. It is unexplored how varying the cation concentration compares to varying the temperature to study melting of immobilized probes.

Melting curves for probes immobilized on surfaces can be generated by two different methods. One of these techniques involves *temporal* temperature gradients and requires special equipment for real-time observation of hybridization or dissociation reactions. Unfortunately, many of these instruments have a relatively low sample throughput. Furthermore, they are often limited with regard to the size of the microarray that can be investigated [13]–[17], The latter limitation can be avoided if expensive scanning microscopy [18] is used for detection. The other method used to create melting curves entails *spatial* temperature gradients [19], [20], in which identical microarrays are usually located in different thermal zones, and each thermal zone provides a point in the corresponding melting curve. The shortcomings of such devices include the following: incompatibility with microarray scanners [19], small heating zones [19], [20], limited temperature control [20], complex fabrication [19], and difficult alignment of DNA microarrays [19], [20]. However, these drawbacks have been eliminated by use of the multi-thermal array washer described in a recent paper [5]. This device has relatively large temperature zones (each ∼24 mm^2^) and is compatible with ordinary glass microscope slides, which allows the use of standard instrumentation for microarray production, hybridization, and detection. The drawbacks of the solution is still that the device is complicated to fabricate and use and is therefore not easily accessible for the average microarray user. Furthermore, due to heat dissipitation, the subarrays need to be fairly small compared to the available space on the slide in order to ensure precise temperatures in each zone.

Here, we describe the design and manufacture of a microfluidic device in which the heating zones in the unit we previously developed [5] were replaced by eight individually controlled stringency zones based on ionic strength. This approach greatly facilitates fabrication and handling of microarrays, because it eliminates the need for incorporation and control of electrical components. The washing station contains eight relatively large zones (each ∼80 mm^2^) for multi-stringency posthybridization washing of microarrays printed on glass microscope slides. The usability of this multi-stringency array washer (MSAW) was demonstrated by genotyping a small cohort of patients for mutations in the human beta-globin gene. Furthermore, the results were compareable with those obtained using the previously developed spatial thermal gradient device [5].

## Materials and Methods

### Design and fabrication of the multi-stringency washer

**Figure 1:**
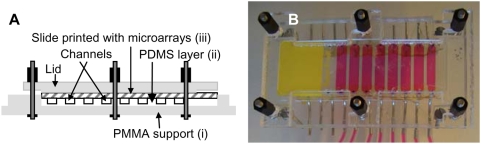
The multi-stringency array washer (MSAW). (A) Schematic drawing of an assembled system viewed from the side. The main parts of the device are shown: (i) the PMMA support; (ii) the PDMS layer, which defines the fluidic channels and the alignment groove for the microscope slide; (iii) the microscope slide printed with identical subarrays that face towards corresponding chambers after the device is sealed. (B) Photograph of the assembled MSAW.

The washer consisted of a solid support made of a 3-mm-thick polymethylmethacrylate (PMMA) sheet on which we mounted an elastic layer containing microfluidic chambers/channels, followed by a component with an alignment groove for a glass microscope slide, and finally a pressure lid (Figure 1). Holes to serve as the in- and outlet of each chamber, as well as for the bolts used to tighten the lid, were drilled in the PMMA support before the elastic layer was inserted. The elastic layer of the polydimethylsiloxane (PDMS) fluidic structure was produced in a separate mold created by micromilling (Folken Industries, Glendale, CA, USA), which defined the washing chambers. The PDMS prepolymer and catalyst (Sylgard 184, Dow Corning, Germany) were mixed and degassed under vacuum for 20 min. Thereafter, the PDMS was added to the mold and degassed for another 20 min before it was cured for 3 h at 80°C and subsequently mounted on the PMMA support plate. Holes for the buffer in- and outlets were prepared by piercing the PDMS in the bottom of the chamber with a sharp needle. The channel connectors were mounted and fixed in place with epoxy glue (Loctite, Dublin, Ireland).

### Preparation of microarrays

Essentially the same probes as described before [5] were employed in this study. Briefly, allele-specific DNA probes were designed for genotyping small genetic variations in the human beta-globin gene (*HBB*). The probes had the variant base/bases positioned as close to the center of the probe as possible, and they contained a poly(T)–poly(C) tag (TC tag) in the 5′ end to facilitate immobilization to agarose films coated slides [21], [22]. Agarose film microarray substrates were prepared as described previously [21], [22]. DNA probes (Table 1) were diluted in MilliQ water to a final concentration of 100 µM and then spotted on the agarose-coated slides by non-contact printing in a NanoPlotter (GeSiM, Großerkmannsdorf, Germany). Spot positions in each subarrray are described by coordinates in Table 1. The probes were immobilized by UV irradiation at 254 nm for 4 min. Thereafter, the slides were washed for 10 min in 0.1×saline sodium citrate (SSC) supplemented with 0.5% sodium dodecyl sulfate (SDS) and then for 10 min in 0.1×SSC to remove unbound probes. Finally, the slides were dried by centrifugation.

**Table 1:** List of primers and probes.

Probe name	Sequence	Position in subarrays (row, col)
**Redesigned shorter probes**		
CD17 mt	TTTTTTTTTTCCCCCCCCCC GTGGGGCTAGGTG	(2,6) (4,6) (6,6) (8,6)
CD17 wt	TTTTTTTTTTCCCCCCCCCCCCCTGTGGGGCAAGGTG	(1,6) (3,6) (5,6) (7,6)
CD24 mt	TTTTTTTTTTCCCCCCCCCCGATGAAGTTGGAGGTGAGGC	(2,8) (4,8) (6,8) (8,8)
CD24 wt	TTTTTTTTTTCCCCCCCCCCATGAAGTTGGTGGTGAGGC	(1,8) (3,8) (5,8) (7,8)
***T*** **m-matched probes**		
CD5 mt	TTTTTTTTTTCCCCCCCCCCGCACCTGACTCGAGGAGAAGT	(2,1) (4,1) (6,1) (8,1)
CD5 wt	TTTTTTTTTTCCCCCCCCCCGCACCTGACTCCTGAGGAGAA	(1,1) (3,1) (5,1) (7,1)
CD8 mt	TTTTTTTTTTCCCCCCCCCCCCTGAGGAGGTCTGCCG	(2,2) (4,2) (6,2) (8,2)
CD8 wt	TTTTTTTTTTCCCCCCCCCCCCTGAGGAGAAGTCTGCCG	(1,2) (3,2) (5,2) (7,2)
CD8-9 mt	TTTTTTTTTTCCCCCCCCCCGAGGAGAAGGTCTGCCGTTAC	(2,3) (4,3) (6,3) (8,3)
CD8-9 wt	TTTTTTTTTTCCCCCCCCCCGAGGAGAAGTCTGCCGTTACTG	(1,3) (3,3) (5,3) (7,3)
CD15 mt	TTTTTTTTTTCCCCCCCCCCACTGCCCTGTAGGGCAAGGT	(2,4) (4,4) (6,4) (8,4)
CD15 wt	TTTTTTTTTTCCCCCCCCCCTGCCCTGTGGGGCAAGG	(1,4) (3,4) (5,4) (7,4)
CD17 mt	TTTTTTTTTTCCCCCCCCCCCCCTGTGGGGCTAGGTGA	(2,5) (4,5) (6,5) (8,5)
CD17 wt	TTTTTTTTTTCCCCCCCCCCCCCTGTGGGGCAAGGTG	(1,5) (3,5) (5,5) (7,5)
CD24 mt	TTTTTTTTTTCCCCCCCCCCAAGTTGGAGGTGAGGCCCT	(2,7) (4,7) (6,7) (8,7)
CD24 wt	TTTTTTTTTTCCCCCCCCCCGAAGTTGGTGGTGAGGCCC	(1,7) (3,7) (5,7) (7,7)
CD27-28 mt	TTTTTTTTTTCCCCCCCCCCGTGAGGCCCCTGGGC	(2,9) (4,9) (6,9) (8,9)
CD27-28 wt	TTTTTTTTTTCCCCCCCCCCGTGAGGCCCTGGGCAG	(1,9) (3,9) (5,9) (7,9)
IVS I +5 mt	TTTTTTTTTTCCCCCCCCCCGGCAGGTTGCTATCAAGGTTACA	(2,10) (4,10) (6,10) (8,10)
IVS I +5 wt	TTTTTTTTTTCCCCCCCCCCGGCAGGTTGGTATCAAGGTTACA	(1,10) (3,10) (5,10) (7,10)
IVS I +6 mt	TTTTTTTTTTCCCCCCCCCCGGCAGGTTGGCATCAAGG	(2,11) (4,11) (6,11) (8,11)
IVS I +6 wt	TTTTTTTTTTCCCCCCCCCCGGCAGGTTGGTATCAAGGTTACA	(1,11) (3,11) (5,11) (7,11)
**PCR primers**		
BCF	AGCAGGGAGGGCAGGAGCCA	
T7-BCR	GAAATTAATACGACTCACTATAGGGAGA-AGAGTCAGTGCCTATCAGAAACCC	

### DNA samples and target preparation

The DNA samples used in this study originated from individuals that were heterozygous (n = 27) or homozygous (n = 4) for a mutation in the *HBB* gene. The original diagnosis was made by measuring an increased level of HbA2 by high-performance liquid chromatography (HPLC), followed by genotyping by automated DNA sequencing. A 300-bp portion of *HBB* containing exon I and the first part of intron I was amplified by PCR using the primer pair BCF and T7-BCR (Table 1). PCR amplification was performed in a total volume of 80 µl containing 1 µM of each primer BCF and T7-BCR, 200 µM of each dNTP, 0.1 U/µl TEMPase Hot Start DNA polymerase (Ampliqon, Bie & Berntsen A/S, Rødovre, Denmark) and 1×TEMPase Buffer II provided with the enzyme. The reverse primer T7-BCR contained a T7 promoter sequence in the 5′ end and thereby served as DNA template for subsequent T7 RNA polymerase amplification. The PCR cycling conditions were 15 min at 95°C followed by 35 amplification cycles at 95°C for 30 s, 60°C for 45 s, 72°C for 1 min, and a final extension at 72°C for 10 min.

Fluorescently marked single-stranded RNA target was produced by T7 *in vitro* transcription (IVT) in an 80 µl reaction mixture containing 8 µl of template DNA, 500 µM of each NTP, 12.5 µM (2.5%) Cy3-CTP (PerkinElmer Life and Analytical Sciences, Boston, MA, USA), 1 U/µl T7 RNA Polymerase-PlusTM (Ambion, Huntingdon, Cambridgeshire, UK) and 1×transcription buffer provided with the enzyme. The reaction was performed at 37°C for 2 h, which resulted in ∼200 ng/µl amplified RNA. Since each slide was hybridized with 80 µl of RNA in a total hybridization volume of 550 µl, the concentration of the 300-nt-long RNA fragment during hybridization was estimated to be 0.3 µM.

### Hybridization and multi-stringency washing

RNA target was diluted to 550 µl in a hybridization buffer with final concentrations of 5×SSC and 0.5% SDS. The hybridization solution was hybridized to a microarray slide using a home built hybridization station for 2 h at 37°C with agitation by bubble movements. After hybridization, the slide was briefly (∼1 min) immersed in 2×phosphate-buffered saline (PBS) and then mounted in the multi-stringency array washer, where it was washed with the eight prepared and preheated buffers for 30 min at 37°C. The eight washing buffers were prepared from stocks of solid sodium dodecyl sulfate (SDS) and 20×SSC buffer. All buffers contained 0.1% SDS and 4.0, 2.0, 1.0, 0.55, 0.25, 0.10, 0.035, or 0.010×SSC, corresponding to 661, 331, 166, 91.6, 42.1, 17.3, 6.58, and 2.45 mM Na^+^, respectively. The buffers were driven through the chambers by an eight-channel peristaltic pump (Watson-Marlow Alitea AB, Stockholm, Sweden) at a rate of ∼0.67 ml/min. The slide was subsequently removed from the array washer and washed for 5 min in 2×PBS at room temperature followed by spin drying.

### Detection and analysis

A Packard Scanarray Lite instrument (Perkin Elmer Life sciences, Boston, MA, USA) was used to scan the microarrays, and all signals were analyzed using GenePix Pro 6.1 (Molecular Devices, Sunnyvale, CA, USA). For each of the eight stringency zones, a normalized ratio for each probe pair was calculated as the signal from the wild-type probe divided by the sum of the signals from the wild-type and mutant probes (S_WT_/(S_WT_+S_MT_)). This means that, at an appropriate stringency during the posthybridization washing step, the normalized ratio of a homozygous wild-type should approach an ideal value of 1.0, which represents a much more intense signal from the wild-type probe than from the mutant probe. Likewise, at any stringency, the ratio of a heterozygote should approach 0.5. This reflects equality in the signal intensities exhibited by the corresponding wild-type and mutant probes. Finally, the ratio of a homozygous mutated sample should approach zero.

## Results

### Dissociation of DNA/RNA duplexes by varying cation concentration

**Figure 2:**
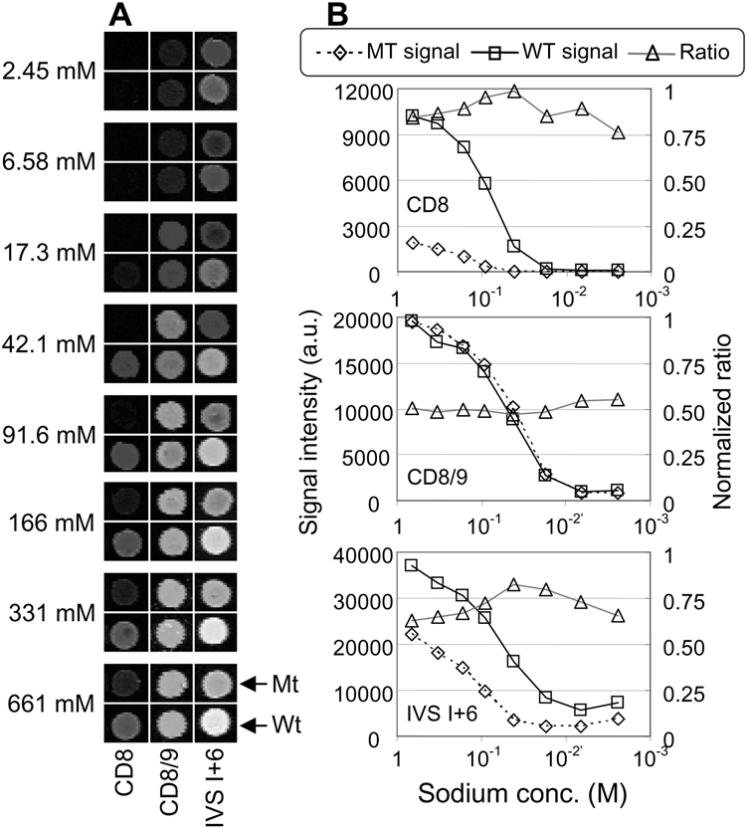
Scanning images of processed arrays and corresponding melting curves. The images show a subset of hybridized probes washed at different sodium concentrations (A). The target material originated from a person heterozygous for the CD8/9 +G mutation. The sodium concentrations in the respective zones are denoted to the left, and the identities of the probes are indicated below the images. Wild-type probes (Wt) for the different mutations and the corresponding mutant probes (Mt) are shown at the bottom and the top of each panel, respectively. The melting curves corresponding to (A) are presented in (B). The wild-type (square) and the mutant (diamond) signal, along with the normalized ratio between those two signals (triangles; see Materials and Methods), are shown for three different mutation sites (CD8 −AA, CD8/9 +G, and IVS I+6 T>C). The graphs are based on the quantified signal intensity (in arbitrary units, a.u.) of the scanning images obtained at the corresponding sodium concentration. For easier comparison with temperature-based dissociation curves obtained previously [5], the X-axis are inverted. All calculations are based on four replicates in each stringency zone. Note that the images in (A) have been changed slightly in terms of contrast and brightness for clarity, whereas the graphs are based on raw signals.

**Figure 3:**
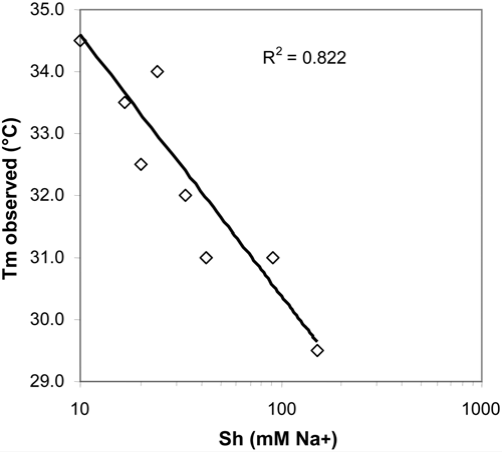
Relationship between measured melting temperature (*T*m) and the sodium concentration at which half of the initial probe signal remained (Sh). The experimentally obtained Sh and *T*m values (*T*m data obtained from reanalyzing the results obtained previously [5]) for each probe pair were plotted against each other, which gave a linear correlation coefficient (R^2^) of 0.822.

To test the performance of the multi-stringency array washer to study melting of hybrids, we used a set of well-characterized probes already known to have similar melting temperatures (±2.8°C) when immobilized on agarose films [5]. A slide hybridized with amplified and labeled RNA, which had been derived from a subject heterozygous at position CD8/9+G, was mounted in the MSAW, and the individual subarrays were washed for 30 min at 37°C with the eight buffers described in Materials and Methods. Figure 2A shows scanning images of the eight subarrays after multi-stringency washing. The signals decreased with increasing stringency, resulting in melting curves that could be used to evaluate assay performance (Fig. 2B). As expected, when analyzing a heterozygous sample, the signal for mutation CD8/9+G was similar for both mutant and wild-type probes over the stringency range, with a normalized ratio of ∼0.5 at all stringencies (Fig. 2, A and B). In contrast, at the remaining mutation sites tested (CD8 and IVS I+6 in Fig. 2, A and B), where the sample was homozygous wild type, we observed stronger signals from wild-type probes than from mutant probes, even at the lowest washing stringencies. Similar melting curves were obtained in our previous investigation [5] when we focused on thermally induced dissociation of duplexes. To be able to compare varying sodium concentrations with varying temperatures in relation to effects on performance of the probe set, we determined the corresponding *T*m values using experimental data generated previously [5] and the sodium concentration at which half of the initial signal remained (Sh) using data in the present study. As illustrated in Figure 3, there was good (logarithmic) linear correlation (R^2^ = 0.82) between the present experimentally determined Sh values and the *T*m values.

### Comparison of different genotyping methods in the MSAW

DNA from a total of 31 subjects was genotyped using the MSAW. Optimal performance of probe pairs was defined as having ratios in the following ranges: 0.7–1 for wild-types, 0.35–0.65 for heterozygotes, and 0–0.3 for mutants. Three different methods of genotyping based on processing in the MSAW were compared, using probes selected to achieve the following: (*i*) similar calculated melting temperature of the entire probe set in a common optimal buffer zone; (*ii*) similar calculated melting temperature of the entire probe set upon washing at different sodium concentrations optimal for each probe pair; (*iii*) optimal performance according to the above-mentioned criteria, regardless of the stringency.

**Figure 4:**
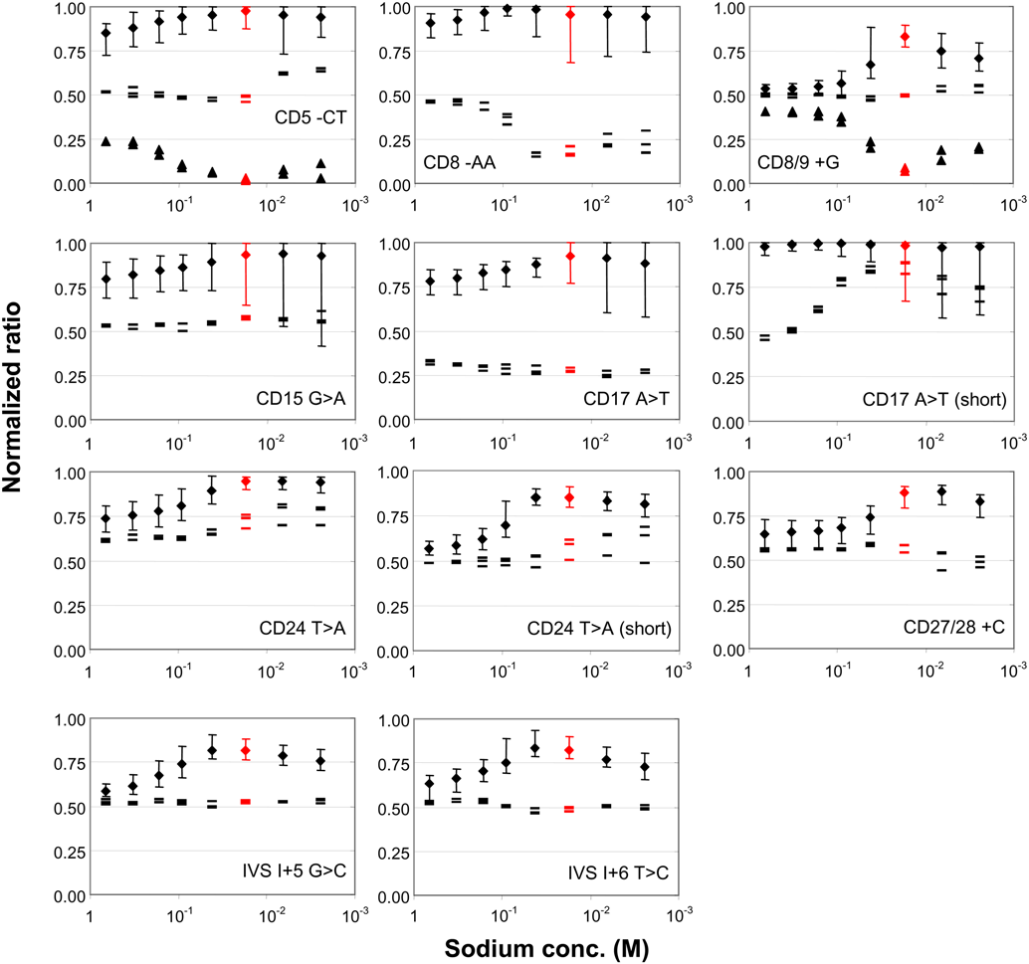
Genotyping of patient material using the MSAW and a *T*m-matched probe set. Thirty-one different samples were individually hybridized to arrays of probes and subsequently processed in the MSAW. For each mutation site, a graph shows the normalized ratios (see Materials and Methods) at the indicated sodium concentrations. The x-axis are inverted to reflect increasing stringency towards the right. Symbols: diamonds, the average value of all samples carrying the wild-type DNA sequence on both alleles (28 samples per mutation, except 26 each for CD5 and CD8/9); error bars, the minimum and maximum observed ratios; dashes, the normalized ratios for heterozygous samples (three samples for each mutation site); triangles, the normalized ratios for homozygous mutations in positions CD5 and CD8/9 (two samples each). The values corresponding to 17.3 mM Na^+^ are shown in red.

**Figure 5:**
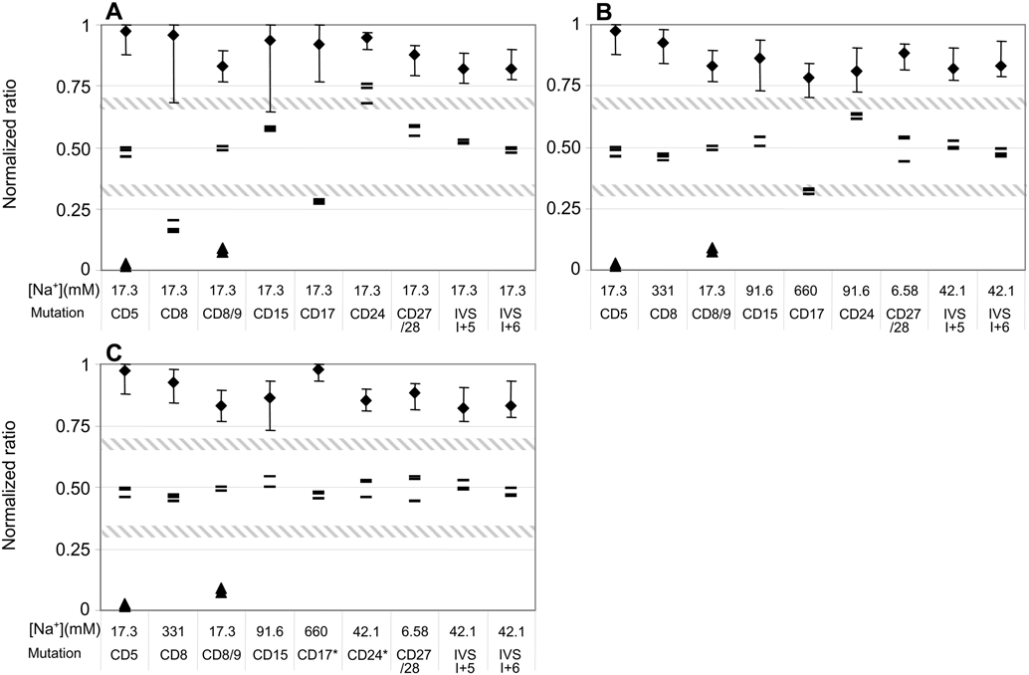
Comparison of different methods of genotyping in the MSAW. We defined probe pair performance as successful when homozygotes had normalized ratios of >0.7 (wild types) or <0.3 (mutants), and heterozygotes had normalized ratios between 0.35 and 0.65; these limits are indicated by the hatched lines in the graphs. (A) *T*_m_-matched probe set in which all probes were washed under the single optimal condition of 17.3 mM sodium. (B) *T*_m_-matched probe set in which each probe pair was washed at its individual optimal sodium concentration, so that the normalized ratio of the heterozygotes was as close to 0.5 as possible, and the separation into genetic classes was as good as could be achieved. (C). A mixed set of probe pairs, all of which originated from the *T*m-matched probe set, except those specific for positions CD17 and CD24, for which we substituted shorter probes (indicatd by asterisks) (see also Table 1). The ratio shown for each probe pair represents the condition that was optimal for clear classification of genotypes, which is indicated by the values given below each mutation. Computation of normalized ratios is explained in the Materials and Methods section, and the symbols used are described in the legend of Figure 4.

Comparing the performance of a Tm matched probe set covering the nine investigated mutation sites revealed that only two conditions (17 or 42 mM Na^+^) allowed unambiguous genotyping of the entire set of samples at a single condition, and, of those two concentrations, 17 mM sodium gave slightly better results (i.e., superior separation of the genetic classes) (Figure 4). There were no misclassifications of the total of 279 genotypings done using 17 mM sodium (Fig. 5A). Nevertheless, three significant problems were encountered when genotyping the probe set at this level of stringency: the probes gave weak signals for CD8-AA (Fig. 2B); separation of CD24 T>A heterozygotes and wild-type ratios was limited, because, compared to the mutant probes, the wild-type probes gave stronger signals for heterozygote samples; the heterozygote ratios for CD17 A>T samples were too low compared to the optimal genotyping criteria given above (Fig. 5A).

Utilizing the MSAW to apply the optimal stringency condition for each pair in the Tm matched probe set (method *ii*) showed that only two of the probe pairs for the nine mutations in the beta-globin gene functioned optimally at a sodium concentration of 17 mM (Figs. 4 and 5B). The probes for the CD8-AA mutation resulted in strong signals (Fig. 2B) and successful separation of homozygotes and heterozygotes at sodium concentrations ranging from 166 to 661 mM (Fig. 4). The probes for CD24T>A still gave poor separation of homozygote wild types and heterozygotes (Fig. 4), whereas the probe pair for CD17A>T resulted in low heterozygote ratios at all stringencies. According to the optimal genotyping criteria mentioned above, neither of the mutations CD17A>T or CD24T>A was genotyped to the fullest by the Tm matched probe set.

To further improve the genotyping assays, the probes for the mutations CD17A>T and CD24T>A were redesigned (MSAW method *iii* above). Compared to the corresponding probes in the initial set, the shorter probes for genotyping CD24T>A had a calculated *T*m that was about 3°C lower, and the mutant probe for CD17A>T was truncated so that its signal would be lowered to better match that emitted by the wild-type probe. Shorter probes for mutation CD24T>A resulted in heterozygote ratios of about 0.5, strong signals, and successful separation of heterozygotes and homozygotes at a sodium concentration of 42 mM (Figs. 4 and 5C). Reducing the length of the mutant probe for CD17A>T resulted in a probe pair that fulfilled the criteria for optimal separation of genotypes at low stringencies (331 and 661 mM Na^+^) (Figs. 4 and 5C).

## Discussion

Prediction of *T*m for solution phase assays has become quite accurate [23], which is useful in the development of homogeneous methods such as multiplex PCR. Since microarray applications comprise highly multiplexed assays that are run under only one condition (similar to multiplex PCR), it is desirable to equalize probe characteristics such as *T*m and ΔG. Accordingly, our goal was to determine whether a sodium concentration gradient could be used instead of a temperature gradient to investigate probe characteristics in order to avoid numerous practical difficulties associated with the thermal approach (see below).

Despite the different mechanisms of applying stringency, we found that a cation gradient behaved very much like a temperature gradient during assay development. This is demonstrated by the observation that the use of different, discrete cation concentrations as stringency modulators provided dissociation curves that were similar to those produced by discrete temperatures (Figs. 2 and 3). Furthermore, the cation gradients were capable of increasing the robustness of the assay in a fashion similar to the results obtained in our previous experiments using temperature gradients [5]. Robustness was augmented by improving classification of genotypes, which was achieved by using a mixture of probes with different optimal operating conditions in the same array. It should also be noted that genotyping of CD24 using the *T*m-matched probe set was equally problematic with the two spatial gradient methods (Fig. 4 and Fig. 5 in [5]), and the solution to that difficulty in both cases was to use shorter probes. The difference between the two approaches was that the sodium gradient was unable to genotype CD17, whereas the thermal gradient could not genotype CD27/28. This disparity might be explained by different sets of spotting and/or oligonucleotide probes, or small variations in the relative density of mutant and wild-type probes. The latter could result in a general upward shift in the CD27/28 genotyping ratio for both heterozygotes and wild-types, as was indeed observed (Fig. 4 and Fig. 3 in [5]), as well as in a general downward shift in the ratio obtained for wild-type and heterozygotes for CD17, as was also detected (Fig. 4). In both cases, values for the heterozygotes were not within the acceptable upper and lower boundaries for that group. Taken together, our findings suggest that gradients of sodium ions (and probably also other cations) can be used in exactly the same way as temperature gradients in the development of array-based assays. In practice, we found that changing the thickness of the Debye layer by modifying the sodium concentration neither complicated nor hindered the assay development.

Generation of cation gradients is not a widespread approach to assay development, despite the fact that this strategy has many advantages over the use of temperature gradients. In contrast to the previously described multi-thermal devices [19], the multi-stringency array washer (MSAW) is not complicated by any electronics or hampered by heat dissipation between chambers/zones in a washing station. Heating zones must be correctly spaced from each other in order to avoid disturbing transfer of heat from one zone to another, and thus the chambers/zones in thermally controlled spatial gradients are small, which means that only relatively small arrays can be investigated. In contrast, using cation concentration to modulate stringency merely requires that individual zones are separated by thin, non-permeable membranes. Accordingly, the MSAW was able to process about threefold larger arrays compared to the multi-thermal counterpart we previously developed [5], which in turn was capable of analyzing about a 75-fold larger array compared to other multi-thermal devices [5], [19], [20]. Another advantage of devices using different stringency buffer zones is that they can be of any size or shape. For example, we found that it was easy to adapt the MSAW for processing of commercial high-density arrays from Agilent (8×15 k slides [24], and therefore after slight modifications, this device can accommodate slides from Nimblegen or Illumina, or any custom-made slide layouts as well. By comparison, it would be a difficult task to process the designated Agilent slides in a spatial temperature gradient, because the individual subarrays in that approach are large and positioned close together.

**Figure 6:**
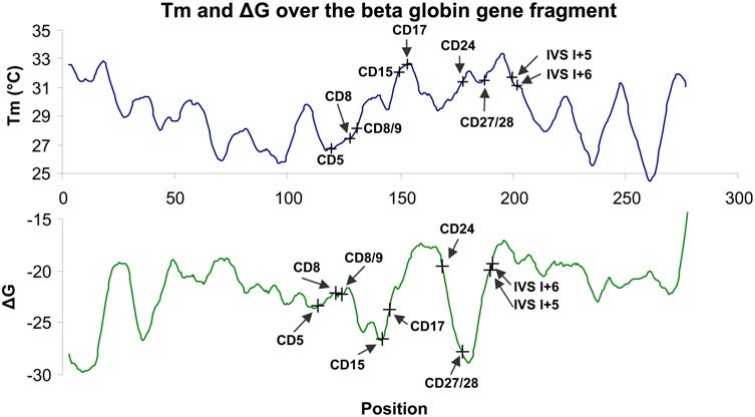
*T*m and ΔG calculated over the beta-globin gene fragment. The graphs are based on the nearest neighbor thermodynamic model for RNA/DNA duplexes, suggested by Sugimoto *et al.* [28]. In this model, each point was initially calculated as the midpoint of a 17-nucleotide window and was subsequently represented as a moving average of five such windows. The graphs illustrate the calculated *T*m (°C) values (top) and ΔG (kcal/mol) values (bottom). The mutation sites investigated in the present study are noted in the graphs.

Allele-specific hybridization (ASH) to DNA microarrays is a powerful method of high-throughput genotyping that is widely used by Affymetrix, as well as many other companies, to analyze single nucleotide polymorphisms (SNPs) [25]–[27]. In contrast, applications such as analysis of point mutations in specific gene segments require an assay format that can handle the large variation in sequence composition that can be encountered in a typical gene segment. Based on the sequence of the beta-globin fragment used in the present study, we used the nearest neighbor parameters of DNA/RNA [28] in 17 nucleotide windows to compute *T*m and ΔG values, and the results indicated variation of about 10°C and 10 kcal/mol respectively over the entire fragment (Fig. 6). This poses a problem, because AT-rich probes must be significantly longer than GC-rich probes to achieve the same *T*m, and thus they are usually less efficient at discriminating mismatch hybrids. Considering that numerous assays require a certain degree of restriction on probe design (e.g., regarding position in the genome), there could be much to gain by employing multiple stringencies, not only while developing the assay, but even during the actual use of the microarray. Especially in diagnostic applications, it would be advantageous to observe a certain characteristic in multiple repeats under different conditions, and thus be able to make a diagnosis based on a trend rather than a single result. For example, we found that even the difficult-to-analyze mutation CD24 could be easily genotyped if we used all eight data points in the gradient instead of just one. As shown in Figure 4, it was hard to differentiate CD24 heterozygotes from homozygotes at low stringency, but separation *gradually* improved as the stringency was raised, as would be expected for a probe pair that is functioning correctly. Thus the use of all data points in the gradient can simplify genotyping of difficult mutations. As for spatial gradients, microarray analysis using temporal gradients can handle *T*m variances in a probe set, since these gradients generate melting curves for matched and mismatched hybrids [13]–[17]. However, recent results have suggested that an isothermal wash is preferable, because detection at different temperatures causes technical problems, such as variation in fluorescence, different washing times, and formation of gas bubbles [16], [29], [30]. Furthermore, short washing cycles are the only feasible solution for temporal gradients, and it is difficult to analyze the data produced by such methodology [31]. The microfluidic devices that create spatial gradients, as presented here and elsewhere [5], [19], [20] were found to have none of the limitations associated with temporal gradients for the following reasons: washing is done for a fixed period of time; scanning is done at one temperature and using dried slides; no photobleaching can occur, because multiple exposures are not necessary. In other words, the MSAW combines the benefits of assaying under many different conditions with the advantages of isothermal washing procedures [31]. Moreover, temporal gradients process only one slide at a time, whereas several MSAW stations can be operated in parallel, which can enable multi-stringency processing of microarrays for end-users who require large sample throughput.

## References

[1] Vainrub A, Pettitt BM (2003). Surface electrostatic effects in oligonucleotide microarrays: control and optimization of binding thermodynamics.. Biopolymers.

[2] Zhang L, Wu C, Carta R, Zhao H (2007). Free energy of DNA duplex formation on short oligonucleotide microarrays.. Nucleic Acids Res.

[3] Marcy Y, Cousin PY, Rattier M, Cerovic G, Escalier G (2008). Innovative integrated system for real-time measurement of hybridization and melting on standard format microarrays.. Biotechniques.

[4] Stromqvist ML, Hopkins K, Liebana E, Brookes AJ (2007). DNA diagnostics by surface-bound melt-curve reactions.. J Mol Diagn.

[5] Petersen J, poulsen L, Petronis S, Birgens H, Dufva M (2008). Use of a multi-thermal washer for DNA microarrays simplifies probe design and gives robust genotyping assays.. Nucleic Acids Res.

[6] Beuvink I, Kolb FA, Budach W, Garnier A, Lange J (2007). A novel microarray approach reveals new tissue-specific signatures of known and predicted mammalian microRNAs.. Nucleic Acids Res.

[7] Castoldi M, Schmidt S, Benes V, Noerholm M, Kulozik AE (2006). A sensitive array for microRNA expression profiling (miChip) based on locked nucleic acids (LNA).. RNA.

[8] Chee M, Yang R, Hubbell E, Berno A, Huang XC (1996). Accessing genetic information with high-density DNA arrays.. Science.

[9] Cheng J, Kapranov P, Drenkow J, Dike S, Brubaker S (2005). Transcriptional maps of 10 human chromosomes at 5-nucleotide resolution.. Science.

[10] Frey BJ, Mohammad N, Morris QD, Zhang W, Robinson MD (2005). Genome-wide analysis of mouse transcripts using exon microarrays and factor graphs.. Nat Genet.

[11] Drobyshev AL, Machka C, Horsch M, Seltmann M, Liebscher V (2003). Specificity assessment from fractionation experiments (SAFE): a novel method to evaluate microarray probe specificity based on hybridisation stringencies.. Nucleic Acids Res.

[12] Vainrub A, Pettitt BM (2002). Coulomb blockage of hybridization in two-dimensional DNA arrays.. Phys Rev E Stat Nonlin Soft Matter Phys.

[13] Anthony RM, Schuitema AR, Chan AB, Boender PJ, Klatser PR (2003). Effect of secondary structure on single nucleotide polymorphism detection with a porous microarray matrix; implications for probe selection.. Biotechniques.

[14] Howell WM, Jobs M, Gyllensten U, Brookes AJ (1999). Dynamic allele-specific hybridization - A new method for scoring single nucleotide polymorphisms.. Nature Biotechnology.

[15] Jobs M, Howell WM, Stromqvist L, Mayr T, Brookes AJ (2003). DASH-2: Flexible, low-cost, and high-throughput SNP genotyping by dynamic allele-specific hybridization on membrane arrays.. Genome Research.

[16] Noerholm M, Bruus H, Jakobsen MH, Telleman P, Ramsing NB (2004). Polymer microfluidic chip for online monitoring of microarray hybridizations.. Lab Chip.

[17] Yershov G, Barsky V, Belgovskiy A, Kirillov E, Kreindlin E (1996). DNA analysis and diagnostics on oligonucleotide microchips.. Proc Natl Acad Sci U S A.

[18] Lee HH, Smoot J, McMurray Z, Stahl DA, Yager P (2006). Recirculating flow accelerates DNA microarray hybridization in a microfluidic device.. Lab Chip.

[19] Kajiyama T, Miyahara Y, Kricka LJ, Wilding P, Graves DJ (2003). Genotyping on a thermal gradient DNA chip.. Genome Research.

[20] Mao HB, Holden MA, You M, Cremer PS (2002). Reusable platforms for high-throughput on-chip temperature gradient assays.. Analytical Chemistry.

[21] Dufva M, Petronis S, Jensen LB, Krag C, Christensen CB (2004). Characterization of an inexpensive, nontoxic, and highly sensitive microarray substrate.. Biotechniques.

[22] Dufva M, Petersen J, Stoltenborg M, Birgens H, Christensen CB (2006). Detection of mutations using microarrays of poly(C)10-poly(T)10 modified DNA probes immobilized on agarose films.. Anal Biochem.

[23] SantaLucia J, Hicks D (2004). The thermodynamics of DNA structural motifs.. Annual Review of Biophysics and Biomolecular Structure.

[24] Poulsen L, Soe MJ, Snakenborg D, Moller LB, Dufva M (2008). Multi-stringency wash of partially hybridized 60-mer probes reveals that the stringency along the probe decreases with distance from the microarray surface.. Nucleic Acids Research.

[25] Wellcome Trust Case Control Consortium (2007). Genome-wide association study of 14,000 cases of seven common diseases and 3,000 shared controls.. Nature.

[26] Matsuzaki H, Dong S, Loi H, Di X, Liu G (2004). Genotyping over 100,000 SNPs on a pair of oligonucleotide arrays.. Nat Methods.

[27] Syvanen AC (2005). Toward genome-wide SNP genotyping.. Nat Genet.

[28] Sugimoto N, Nakano S, Katoh M, Matsumura A, Nakamuta H (1995). Thermodynamic Parameters to Predict Stability of Rna/Dna Hybrid Duplexes.. Biochemistry.

[29] Liu WT, Wu JH, Li ES, Selamat ES (2005). Emission characteristics of fluorescent labels with respect to temperature changes and subsequent effects on DNA microchip studies.. Appl Environ Microbiol.

[30] Pozhitkov A, Chernov B, Yershov G, Noble PA (2005). Evaluation of gel-pad oligonucleotide microarray technology by using artificial neural networks.. Appl Environ Microbiol.

[31] Pozhitkov AE, Stedtfeld RD, Hashsham SA, Noble PA (2007). Revision of the nonequilibrium thermal dissociation and stringent washing approaches for identification of mixed nucleic acid targets by microarrays.. Nucleic Acids Res.

